# Reverse Effect of Mammalian Hypocalcemic Cortisol in Fish: Cortisol Stimulates Ca^2+^ Uptake via Glucocorticoid Receptor-Mediated Vitamin D_3_ Metabolism

**DOI:** 10.1371/journal.pone.0023689

**Published:** 2011-08-24

**Authors:** Chia-Hao Lin, I-Lun Tsai, Che-Hsien Su, Deng-Yu Tseng, Pung-Pung Hwang

**Affiliations:** 1 Graduate Institute of Life Sciences, National Defense Medical Center, Taipei, Taiwan, Republic of China; 2 Institute of Cellular and Organismic Biology, Academia Sinica, Taipei, Taiwan, Republic of China; 3 Department of Aquaculture, National Pingtung University of Science and Technology, Pingtung, Taiwan, Republic of China; 4 Institute of Fishery Science, National Taiwan University, Taipei, Taiwan, Republic of China; 5 Department of Biological Sciences and Technology, National University of Tainan, Tainan, Republic of China; National Institute on Aging Intramural Research Program, United States of America

## Abstract

Cortisol was reported to downregulate body-fluid Ca^2+^ levels in mammals but was proposed to show hypercalcemic effects in teleostean fish. Fish, unlike terrestrial vertebrates, obtain Ca^2+^ from the environment mainly via the gills and skin rather than by dietary means, and have to regulate the Ca^2+^ uptake functions to cope with fluctuating Ca^2+^ levels in aquatic environments. Cortisol was previously found to regulate Ca^2+^ uptake in fish; however, the molecular mechanism behind this is largely unclear. Zebrafish were used as a model to explore this issue. Acclimation to low-Ca^2+^ fresh water stimulated Ca^2+^ influx and expression of *epithelial calcium channel* (*ecac*), *11β-hydroxylase* and the *glucocorticoid receptor* (*gr*). Exogenous cortisol increased Ca^2+^ influx and the expressions of *ecac* and *hydroxysteroid 11-beta dehydrogenase 2 (hsd11b2)*, but downregulated *11β-hydroxylase* and the *gr* with no effects on other Ca^2+^ transporters or the *mineralocorticoid receptor* (*mr*). Morpholino knockdown of the GR, but not the MR, was found to impair zebrafish Ca^2+^ uptake function by inhibiting the *ecac* expression. To further explore the regulatory mechanism of cortisol in Ca^2+^ uptake, the involvement of vitamin D_3_ was analyzed. Cortisol stimulated expressions of *vitamin D-25hydroxylase* (*cyp27a1*), *cyp27a1 like* (*cyp27a1l*), *1α-OHase* (*cyp27b1*) at 3 dpf through GR, the first time to demonstrate the relationship between cortisol and vitamin D_3_ in fish. In conclusion, cortisol stimulates *ecac* expression to enhance Ca^2+^ uptake functions, and this control pathway is suggested to be mediated by the GR. Lastly, cortisol also could mediate vitamin D_3_ signaling to stimulate Ca^2+^ uptake in zebrafish.

## Introduction

Corticosteroids (CSs) are primarily synthesized from cholesterol through a series of reactions. CSs, which consist of glucocorticoids (GCs) and mineralocorticoids (MCs), are vital hormones for mammals, and are involved in regulating the osmolality and ion levels, body fluids, energy metabolism, respiration, and immune reactions [1], [2]. GCs are efficient treatment for asthma, rheumatoid arthritis, and atopic dermatitis because they can reduce immune responses; however, several systemic side-effects including osteoporosis are induced. An imbalance of Ca^2+^ handling is an important factor causing osteoporosis [3]. Regulation of Ca^2+^ absorption and emission is closely associated with the bone structure, and GC was reported to cause malabsorption and malemission of Ca^2+^ in the intestines and kidneys [2]–[4]. In mammals, GC was proposed to downregulate Ca^2+^ levels of body fluids through modulating the renal and duodenal expressions of TRPV6 and calbindin-D_9_K [5], [6].

CS is synthesized in the adrenal cortex of mammals, but in the interrenal tissue of the head kidneys in teleosts. Physiological functions of CSs in teleosts are similar to those in mammals, and CS signaling is also mediated by the GC receptor (GR) and MC receptor (MR), which are ligand-activated transcription factors [7], [8]. Both the GR and MR can bind the GC-responsive element (GRE) of the gene promoter and form GR-GR, MR-MR, and MR-GR dimers [9]. In CS synthesis, 11β-hydroxylase (CYP11B1) and aldosterone synthase (CYP11B2) are enzymes in the final step of the synthesis of cortisol and aldosterone, respectively. Teleosts may lack aldosterone synthase, and therefore cortisol is the main CS hormone in teleosts [10], [11]. Some in vitro studies demonstrated that cortisol stimulated the transcriptional activity in mammalian cell lines transiently transfected with an expression construct containing a fish GR or MR and a reporter plasmid containing multiple GREs, implying that both teleostean CS receptors can be bound by cortisol with different affinities [12]–[15]. Based on those results, cortisol was suggested to have both GC and MC functions through different CS receptors, GR or MR, in teleosts; however, very few studies have investigated if the GR, MR, or both are involved in specific physiological processes in teleosts. In a recent study on Atlantic salmon, the GR and MR were found to differentially mediate the stimulation of various ion transporters in the gills during acclimation to salinity changes [16].

Vitamin D_3_ was a vital calcitrophic endocrine to regulate Ca^2+^ homeostasis in vertebrates. Liver vitamin D-25hydroxylase (CYP27A1) converts vitamin D_3_ precursor to 25-hydroxyvitamin D_3_ (25(OH)D_3_), which is then converted to 1,25-dihydroxytamin D_3_ (1,25 (OH)_2_D_3_), the active form of the vitamin D_3_, by renal 1α-OHase (CYP27B1) [17]. Vitamin D_3_ spreads its function through its receptor, vitamin D_3_ receptor (VDR). VDR is a ligand-active transcription factor, and duodenal *trpv6* (*ecac*) is the target of the vitamin D_3_-VDR complex in mammals [18]. Stimulation of the duodenal *trpv6* expression by vitamin D_3_ is one of important pathways to enhance Ca^2+^ uptake in mammals [19]. In fish, vitamin D_3_ had been demonstrated to elevate serum Ca^2+^ level [20], [21] .Vitamin D_3_ was also proposed to be associated with Ca^2+^ transport in the gills based on the vitamin D_3_ deficiency-induced impairment of growth and mineralization in the fish skeleton [22], [23]. Indeed, changes in the plasma levels of 1,25 (OH)_2_D_3_ or mRNA expression of gill/kidney VDR have been observed in salmon undergoing smoltification and migrating from freshwater (low Ca^2+^ concentrations) to seawater (high Ca^2+^ concentrations), suggesting that synthesis of the sterol and its receptor might be regulated depending upon ambient Ca^2+^ concentrations [23]. In mammals, glucocorticoid has been well documented to affect vitamin D_3_ metabolism although the actions varied depending on species [24]–[29]. This suggests a possible association between glucocorticoid, vitamin D_3_ and Ca^2+^ homeostasis in mammals; however, it is unknown whether this connection is also developed in fish.

In teleosts, cortisol is well demonstrated to regulate the mechanisms of ionic and osmotic balance, but only few studies investigated the role of cortisol in controlling transepithelial Ca^2+^ transport [30], [31]. Teleostean fish, unlike terrestrial vertebrates, obtain Ca^2+^ mainly via absorption from the environment rather than by dietary means. In adult fish, the predominant route of Ca^2+^ entry from the environment is across the gill epithelium while in larvae, the body skin is the major route of Ca^2+^ uptake before full development of the gills occurs [32], [33]. The Ca^2+^ uptake function is well regulated for maintaining the internal Ca^2+^ homeostasis during acclimation to aquatic environments with fluctuating Ca^2+^ levels (<0.01 mM in soft fresh water to >10 mM in seawater) [30], [34]. According to the current model in mammals and teleosts, active transcellular Ca^2+^ transport is carried out through the operation of apical epithelial Ca^2+^ channels (ECaC, TRPV5, and/or TRPV6), and basolateral plasma membrane Ca^2+^-ATPase (PMCA) and the Na^+^/Ca^2+^ exchanger (NCX) [34], [35]. Exposure of rainbow trout to a reduced ambient calcium level induces a rapid increase in systemic cortisol levels [36], and exogenous cortisol can stimulate branchial ECaC mRNA and protein expressions [37]. In an experiment with cultured gills, cortisol was also found to enhance transepithelial Ca^2+^ transport [38]. Those studies suggested a role of cortisol in control of Ca^2+^ uptake in teleosts; however, it is still unclear if cortisol controls only ECaCs or other Ca^2+^ transporters (NCX and PMCA), and if this control pathway is mediated by the GR, MR, or both. Elucidation of these issues would enhance our understanding of the molecular physiological mechanisms of cortisol's control of epithelial Ca^2+^ transport, an essential component associated with bone structure and formation in vertebrates.

Recently, zebrafish have become a model for research on ion regulation and related endocrine controls due to the well-developed genetic database and applicability of various molecular physiological approaches [32], [39]. In zebrafish gills and skin (in embryonic stages), a specific ionocyte type that expresses ECaC, PMCA2, and NCX1b was identified to be responsible for transepithelial Ca^2+^ uptake function [32], [33], [39]–[41], providing a suitable model to further explore cortisol's control of Ca^2+^ uptake mechanisms. The present study attempted to address 3 specific questions: (I) Does cortisol control zebrafish Ca^2+^ uptake function by regulating the *ecac*, *ncx1b*, and/or *pmca2*? (II) Does cortisol control zebrafish Ca^2+^ uptake function through mediation by the GR, MR, or both receptors? (III) Does cortisol control zebrafish Ca^2+^ uptake function by regulating vital calcitrophic endocrine suchlike vitamin D_3_? The effects of environmental Ca^2+^ levels and exogenous cortisol on Ca^2+^ contents and influx, the mRNA expressions of Ca^2+^ transporters (*ecac, ncx1b, and pmca2*), the steroidogenesis enzymes *11β-hydroxylase* and *hydroxysteroid 11-beta dehydrogenase 2 (hsd11b2)*, CS receptors (*gr* and *mr*) and vitamin D_3_-related genes (*vdra*, *vdrb*, *cyp27a1*, *cyp27a1l* and *cyp27b1*) were investigated. Moreover, effects of knockdown of the GR or MR on Ca^2+^ contents and influx, and the expression of Ca^2+^ transporters and vitamin D_3_-related genes in zebrafish embryos were examined.

## Methods

### Experiment animals

Zebrafish (*Danio rerio*) were kept in local tap water ([Ca^2+^] = 0.2 mM) at 28.5°C under a 14∶10-h light-dark photoperiod at the Institute of Cellular and Organismic Biology, Academia Sinica, Taipei, Taiwan. Experimental protocols were approved by the Academia Sinica Institutional Animal Care and Utilization Committee (approval no.: RFIZOOHP220782).

### Acclimation experiments

Artificial fresh waters with high- (2 mM) and low-Ca^2+^ (0.02 mM) levels were prepared with double-deionized water (model Milli-RO60; Millipore, Billerica, MA, USA) supplemented with adequate CaSO_4_·2H_2_O, MgSO_4_·7H_2_O, NaCl, K_2_HPO_4_, and KH_2_PO_4_. Ca^2+^ concentrations (total Ca^2+^ levels measured by absorption spectrophotometry) of the high- and low-Ca^2+^ media were 2 and 0.02 mM, respectively, but the other ion concentrations of the 3 media were the same ([Na^+^], 0.5 mM; [Mg^2+^], 0.16 mM; and [K^+^], 0.3 mM) as those in local tap water. Variations in ion concentrations were maintained within 10% of the predicted values. Fertilized zebrafish eggs were transferred to high- and low- Ca^2+^ media, respectively, and incubated thereafter until sampling at 3 d post-fertilization (dpf). The sampling time in this study was based previously [42].

### Cortisol incubation experiments

For cortisol incubation experiments, we based cortisol dosage from previous study [43]. Cortisol (hydrocortisone, H4881, Sigma Chemical Co., St Louis, MO, USA) was dissolved in local tap water at 0 (control), 20, and 40 mg/l. Zebrafish embryos were incubated in the cortisol media immediately after fertilization, and were sampled at 1 or 3 dpf for the subsequent analysis. The incubation media were changed with new cortisol solution every day to maintain constant levels of cortisol. During incubation, neither significant mortality nor abnormal behavior was found.

### Whole-body Ca^2+^ content

Zebrafish embryos were anesthetized with MS-222 (Sigma) and then briefly rinsed in deionized water. 30 individuals were pooled as 1 sample. HNO_3_ (13.1 N) was added to samples for digestion at 60°C overnight. Digested solutions were diluted with double-deionized water, and the total Ca^2+^ content was measured with a Z-8000 atomic absorption spectrophotometer (Hitachi, Tokyo, Japan). Standard solutions (Merck, Darmstadt, Germany) were used to make the standard curves.

### Whole-body Ca^2+^ influx

By following previously described methods [44] with some modifications, zebrafish embryos were dechorionated, rinsed briefly in deionized water, and then transferred to 2 ml of ^45^Ca^2+^ (Amersham, Piscataway, NJ; with a final working specific activity of 1∼2 mCi/mmol)-containing medium for a subsequent 4-h incubation. After incubation, embryos were washed several times in isotope-free water medium. Six embryos were pooled into 1 vial, anesthetized with MS-222, and digested with tissue solubilizer (Solvable; Packard, Meriden, CT, USA) at 60°C for 8 h. The digested solutions were supplemented with counting solution (Ultima Gold; Packard), and the radioactivities of the solutions were counted with a liquid scintillation beta counter (LS6500; Beckman, Fullerton, CA, USA). The Ca^2+^ influx was calculated using the following formula: *J*_in_ = Q_embryo_ X_out_^−1^ *t* ^−1^ W^−1^; where *J*_in_ is the influx (pmol·mg^−1^·h^−1^), Q_embryo_ is the radioactivity of the embryo (cpm per individual) at the end of incubation, X_out_ is the specific activity of the incubation medium (cpm/pmol), *t* is the incubation time (h), and W is the average body wet weight of different-stage embryos (mg).

### RNA extraction

After anesthetized with 0.03% MS222, appropriate amounts of zebrafish tissues or embryos were collected and homogenized in 1 ml Trizol reagent (Invitrogen, Carlsbad, CA, USA), then mixed with 0.2 ml chloroform, and thoroughly shaken. After centrifugation at 4°C and 12,000×*g* for 30 min, the supernatants were obtained. The samples were then mixed with an equal volume of isopropanol. Pellets were precipitated by centrifugation at 4°C and 12,000×*g* for 30 min, washed with 70% alcohol, and stored at −20°C until use.

### Reverse-transcription polymerase chain reaction (RT-PCR) analysis

For complementary (c)DNA synthesis, 1∼5 µg of total RNA was reverse-transcribed in a final volume 20 µl containing 0.5 mM dNTPs, 2.5 µM oligo (dT)_20_, 250 ng random primers, 5 mM dithiothreitol, 40 units RNase inhibitor, and 200 units Superscript RT (Invitrogen) for 1 h at 50°C followed a 70°C incubation for 15 min. For PCR amplification, 2 µl cDNA was used as template in a 50-µL final reaction volume containing 0.25 mM dNTPs, 2.5 units Taq polymerase (Takara, Shiga, Japan), and 0.2 µM of each primer (Table S1). 30 cycles were performed for each reaction. All amplicons were sequenced to ensure that the PCR products were the desired gene fragments.

### Quantitative real-time PCR (qPCR)

qPCR was performed with a LightCycler real-time PCR system (Roche, Penzberg, Germany) in a final volume of 10 µl, containing 5 µl 2× SYBR Green I Master (Roche Applied System), 300 nM of the primer pairs, and 20∼30 ng cDNA. The standard curve for each gene was checked in a linear range with β-actin as an internal control. The primer sets for the qPCR are shown in Table S2.

### In situ hybridization

Zebrafish *ecac* or *gr* Fragments were obtained by PCR and inserted into the pGEM-T easy vector (Promega, Madison, WI, USA). The inserted fragments were amplified with the T7 and SP6 primers by PCR, and the products as templates were used for the in vitro transcription with T7 and SP6 RNA polymerase (Roche) in the presence of digoxigenin (DIG)-UTP (Roche) to, respectively, synthesize sense and anti-sense probes. Zebrafish embryos were anesthetized on ice and fixed with 4% paraformaldehyde (PFA) in a phosphate-buffered saline (PBS; 1.4 mM NaCl, 0.2 mM KCl, 0.1 mM Na_2_HPO_4_, and 0.002 mM KH_2_PO_4_; pH 7.4) solution at 4°C overnight. To do in situ hybrizization, we followed previously [40]. For the quantification of density, eight areas (85×80 µm^2^ each) on the yolk sac surface of an embryo were chosen for counting.

### Organ culture

Adult fish were anesthetized with 0.03% MS222 and then gills were dissected and directly transferred to the pre-incubation DMEM medium (Invitrogen) containing 50 mg/ml of penicillin (Invitrogen), 50 µg/ml of streptomycin (Invitrogen) and 20% Fetal Bovine Serum (Invitrogen). Individual gill arches were carefully separated from the whole gill structure. Each gill arch was cut lengthwise, and the cut filament was designated as one sample to be incubated in a well (96-well). The cut gill filaments were incubated with the freshly prepared pre-incubation medium (control group) and the DMEM with supplementary 20 mg/l cortisol, respectively. The media were freshly prepared and replaced twice per day. Organ culture was carried out at 28°C for 1 d in 96-well culture plates in a humidified chamber supplied with 95% O_2_ and 5% CO_2_.

### Morpholino oligonucleotide (MO) knockdown and rescue

The zebrafish MR MO (5′-GTATCTTTTAGTCTCCAT-3′) and GR MO (5′-TCCAGTCCTCCTTGATCCAT-3′) were prepared with 1× Danieau solution (58 mM NaCl, 0.7 mM KCl, 0.4 mM MgSO_4_, 0.6 mM Ca(NO_3_)_2_, and 5.0 mM HEPES; pH 7.6). A standard control MO (5′-CCTCTTACCTCAGTTACAATTTATA-3′) was used as the control. To confirm MO specificity, fragments of the GR and MR containing the MO-targeted sequences were PCR-amplified with gene-specific primers (Table S3) and then cloned into the pCS2+GFP XLT vector, and the expression constructs were linearized to synthesize capped mRNA (cRNA) using an SP6 message RNA polymerase kit (Ambion, Austin, TX, USA). To confirm safety and efficiency of MOs and cRNA, we tested to inject various dosages of MOs and cRNA. Finally, we choose 2 ng/embryo (for MO) and 300 pg/embryo (for cRNA) to inject. Under these dosages, neither significant mortality nor abnormal behavior was found. The MO (2 ng/embryo) and/or cRNA (300 pg/embryo) were injected into embryos at the 1∼2 cell stage using an IM-300 microinjector system (Narishige Scientific Instrument Laboratory, Tokyo, Japan). Green fluorescent protein (GFP) signals in 1-dpf embryos were observed by fluorescence microscopy (Axioplan 2 Imaging; Carl Zeiss, Oberkochen, Germany). MO-injected embryos at 1 or 3 dpf were sampled for subsequent analyses.

To rescue the defects caused by the MO, a full-length GR was PCR-amplified with a specific primer (Table S3) and cloned into the pCS2+ vector, and the construct was used to synthesize cRNA. Another GR MO, GR-SB MO, was designed at an intron-exon boundary by following a previous study [45], and this MO could only block endogenous GR translation. The full-length GR cRNA(300 pg/embryo) and GR-SB MO (5′-CTGCTTCATGTATTTTAGG-3′; 2 ng/embryo) were injected into embryos at the 1∼2 cell stage, and embryos were sampled at 3 dpf.

### Western blot analysis

Thirty embryos were pooled as one sample and homogenized. Protein of 50 mg/well was loaded to a 10% SDS-PAGE at 100 V for 2 h. After separation, proteins were transferred onto polyvinylidene difluoride membrane (Millipore) at 100 V for 2 h. After being blocked for 1.5 h in 5% nonfat milk, the blots were incubated with GR (Santa Cruz Biotechnology) or MR polyclonal antibody (Abcam) overnight 4°C, diluted 1∶500 and with an alkaline-phos-phatase-conjugated goat anti-rabbit IgG (diluted 1∶2500, room tem-perature; Jackson Laboratories) for another 2 h. The blots were developed with 5-bromo-4-chloro-3-indolylphosphate/nitro-blue tetrazolium.

### Cryosectioning

Fresh zebrafish gills were fixed with 4% PFA at 4°C for 3 h and then immersed serially in PBS containing 5, 10, and 20% sucrose for 15 min at room temperature. Finally, gills were soaked in a mixed PBS solution (OCT compound: 20% sucrose at 1∶2) overnight and then embedded with OCT compound embedding medium (Sakura, Tokyo, Japan) at 20°C. Cryosections at 6 µm were made with a cryostat (CM 1900; Leica, Heidelberg, Germany), and these were placed onto poly-L-lysine-coated slides (EMS, Hatfield, PA).

### Immunocytochemistry

Prepared slides were rinsed in PBS and blocked with 3% BSA for 30 min. Afterward, the slides were first incubated with an α5 monoclonal antibody against the α-subunit of the avian Na,K-ATPase (NKA) (Hybridoma Bank, University of Iowa, Ames, IA; 1∶600 dilution) overnight at 4°C. The slides were washed twice with PBS and incubated with an Alexa Fluor 568 goat anti-mouse IgG antibody (Molecular Probes, Carlsbad, CA; 1∶200 diluted with PBS) for 2 h at room temperature. Images were acquired with a Leica TCS-NT confocal laser scanning microscope (Leica) or an Axioplan 2 imaging microscope.

### Potential regulatory elements upstream of the zebrafish *ecac* gene

The zebrafish *ecac* genomic sequence was obtained from a zebrafish genome database (http://www.ncbi.nlm.nih.gov/projects/genome/guide/zebrafish/). Potential regulatory elements upstream of the *ecac* gene were predicted by Genomatix MatInspector (http://www.genomatix.de).

### Statistical analysis

Data are presented as the mean±SD and were analyzed by one-way ANOVA and Student's *t*-test.

## Results

### mRNA expressions of *mr* and *gr*

**Figure 1:**
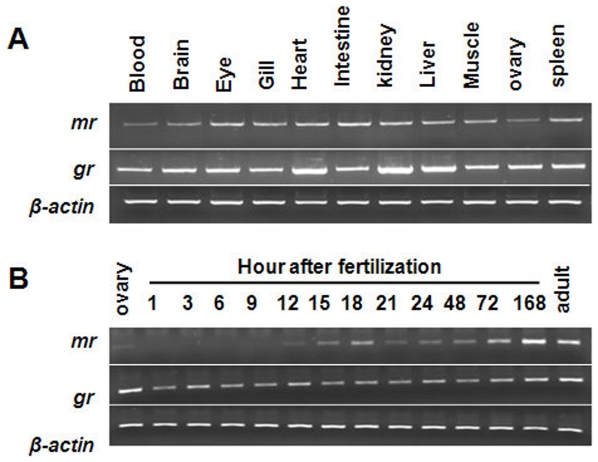
Zebrafish *mr* and *gr* expression profiles. Determined by RT-PCR, *mr* and *gr* mRNA in various tissues of adults (A), and during developmental stages of embryos (B). *β-actin* was used as the internal control.

Both *mr* and *gr* mRNAs were universally expressed in all tissues studied (Fig. 1A). In developing embryos, mRNA expression of the *gr* was first detected at 1 h post-fertilization (hpf) and throughout development; however, *mr* mRNA only began to be expressed at 12 hpf (Fig. 1B).

### Ca^2+^ incubation on Ca^2+^ influx and Ca^2+^ -related genes

**Figure 2:**
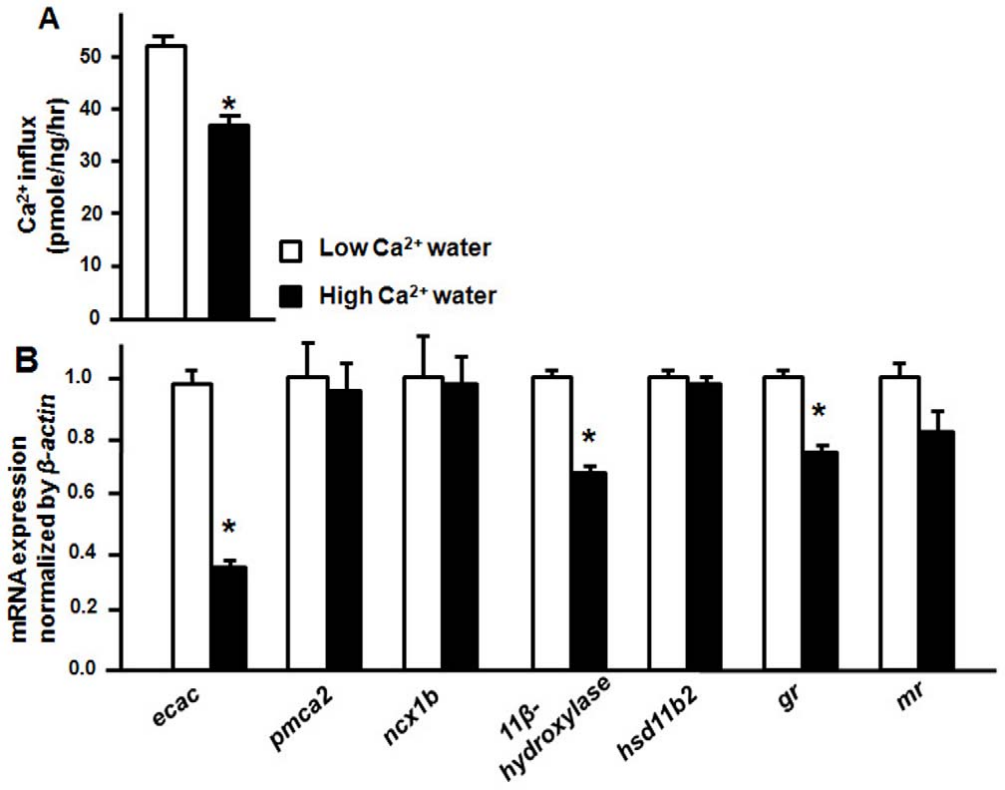
Ca^2+^ influx and gene expressions of Ca^2+^ regulation-related genes. Ca^2+^ influx (A) and mRNA expression (B) of 3-dpf zebrafish embryos acclimated to low- (0.02 mM Ca^2+^) or high-Ca^2+^ (2.00 mM Ca^2+^) artificial fresh water. mRNA expression analyzed by qPCR and values were normalized to *β-actin*. Values are the mean ± SEM (*n* = 4∼6). *Significant difference (Student's *t-test*, *p*<0.05).

After acclimation to artificial fresh water containing different levels of Ca^2+^ for 3 d, zebrafish Ca^2+^ influx was significantly stimulated by low-Ca^2+^ water (Fig. 2A). Similarly, *ecac*, *11β-hydroxylase* and *gr* mRNA expressions were also significantly stimulated by low-Ca^2+^ water (Fig. 2B). On the contrary, *pmca2*, *ncx1b*, *hsd11b2*, and *mr* mRNA expressions were not affected by environmental Ca^2+^ levels (Fig. 2B).

### Exogenous cortisol on Ca^2+^ influx/contents and mRNA expressions of Ca^2+^-related target genes

**Figure 3:**
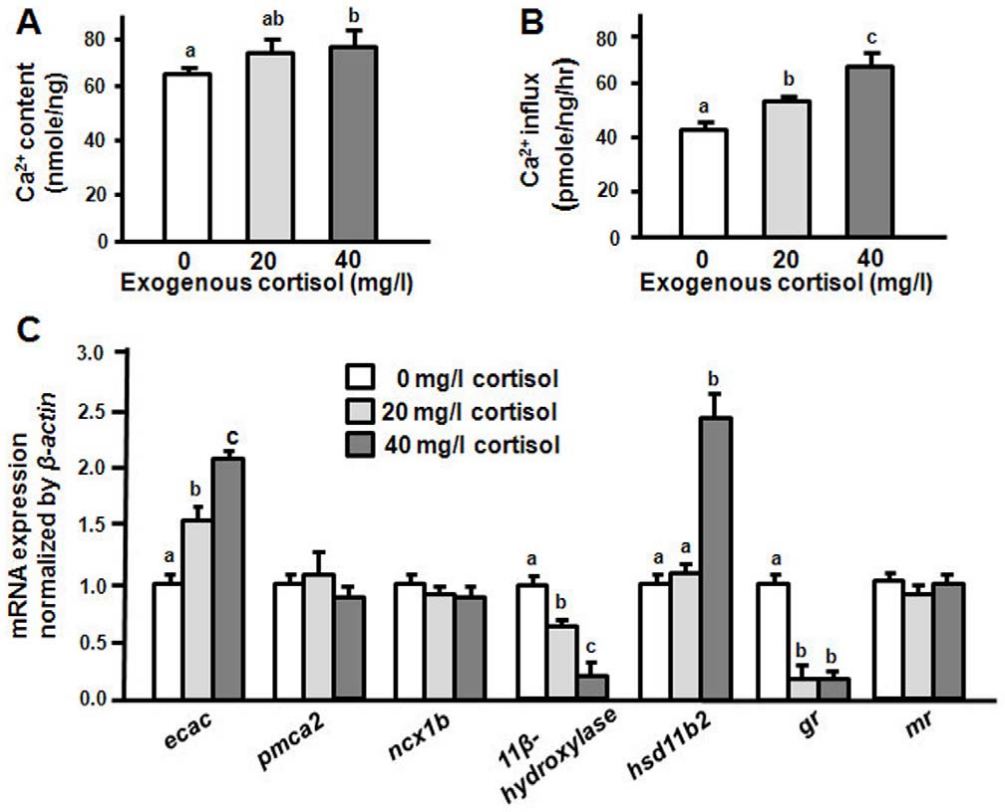
Effects of exogenous cortisol in 3-dpf zebrafish embryos. Ca^2+^ content (A), Ca^2+^ influx (B) and mRNA expressions (C). mRNA expressions were analyzed by qPCR, and values were normalized to *β-actin*. ^abc^Indicate a significant difference (*p*<0.05) using Tukey's multiple-comparison test following one-way ANOVA. Value are the mean ± SEM (*n* = 6 or 7).

**Figure 4:**
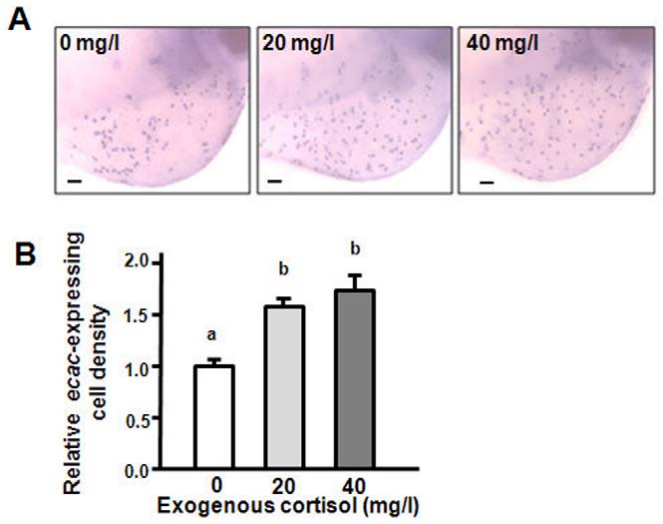
Effects of exogenous cortisol on *ecac*-expressing cells in 3-dpf zebrafish embryos. In situ hybridization analysis indicated *ecac* signals (A) and density of *ecac*-expressing cells (B). ^abc^Indicate a significant difference (*p*<0.05) using Tukey's multiple-comparison test following one-way ANOVA. Value are the mean ± SEM (*n* = 6 or 7). *Scale bar* 100 µm.

To test the hypothesis of whether cortisol can affect Ca^2+^ uptake, zebrafish embryos at the 1∼2-cell stage were treated with exogenous cortisol for 3 d. Incubation with exogenous cortisol caused dose-dependent effects on both Ca^2+^ contents and influx in 3-dpf zebrafish embryos. Compared to the control group (0 mg/l), cortisol-treated groups (20 and 40 mg/l) showed significant increases in Ca^2+^ content and influx (Fig. 3A, B). The qPCR revealed differential effects of exogenous cortisol on the mRNA expressions of Ca^2+^ transporters. mRNA expressions of zebrafish *ncx1b* and *pmca2* were not affected by cortisol treatment (Fig. 3C); however, that of *ecac* was significantly upregulated by exogenous cortisol in a dose-dependent pattern (Fig. 3C). Furthermore, exogenous cortisol was also used to treat cultured gills. Similarly, *ecac* mRNA expression was affected by exogenous cortisol in gills, but *ncx1b* and *pmca2* were not affected (Fig. S1). To support the data (Fig. 3C) of qPCR analyses for Ca^2+^ transporter expressions, in situ hybridization of *ecac* was conducted in the embryos treated with cortisol. As shown in Fig. 4A and B, exogenous cortisol significantly stimulated the density of *ecac* -expressing cells in 3-dpf zebrafish embryos. Exogenous cortisol also caused differential effects on mRNA expressions of *11β-hydroxylase*, *hsd11b2*, *gr*, and *mr* in zebrafish embryos. According to the qPCR in 3-dpf embryos, exogenous cortisol significantly inhibited mRNA expressions of *11β-hydroxylase* (in a dose-dependent manner) and *gr* and stimulated *hsd11b2*, but did not affect that of the *mr* (Fig. 3C).

### Loss-of-function effects on Ca^2+^ contents/influx/transporters and density of *ecac*-expressing cells in zebrafish embryos

**Figure 5:**
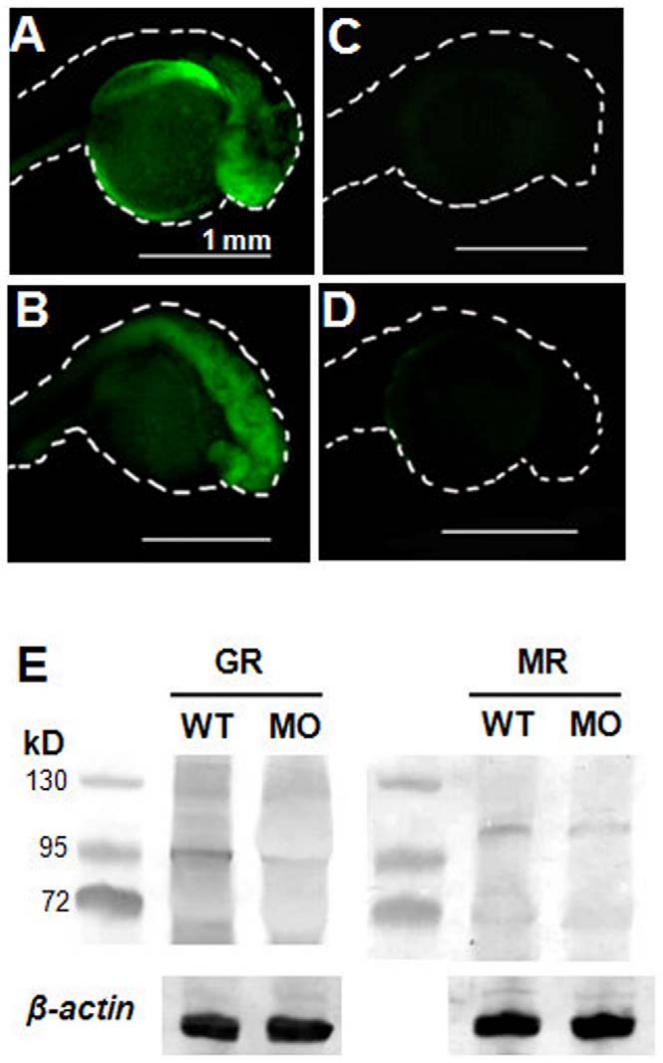
Specificity and effectiveness of MR MO and GR MO. MR and GR cRNA (with GFP fusion) were injected into embryos respectively (A, B), and embryos coinjection of MR/GR MO with cRNA (C, D). Western blot were used to detect GR and MR protein expressions in wild type (WT) and the MO-injected embryos at 3 dpf (E).

To block the endogenous cortisol signaling pathway, MR and GR MOs were used to respectively inhibit translation of zebrafish GR and MR. The specificity and effectiveness of the MR and GR MOs were respectively confirmed by co-injection with zebrafish MR or GR cRNAs. Zebrafish embryos injected with only cRNAs (with GFP fusion) revealed signals of GFP translation (Fig. 5A, B), confirming the translation of MR and GR cRNAs. On the other hand, embryos co-injected with the MR (or GR) MO and MR (or GR) cRNA with GFP showed no green fluorescence (Fig. 5C, D), indicating that the MO specifically and effectively knocked-down the translation of MR (or GR) mRNA. In addition, Western blot was also used to further demonstrate MO specificity. As a result, GR or MR MO was found to specifically downregulate GR or MR protein level in 3-dpf zebrafish embryos (Fig. 5E).

**Figure 6:**
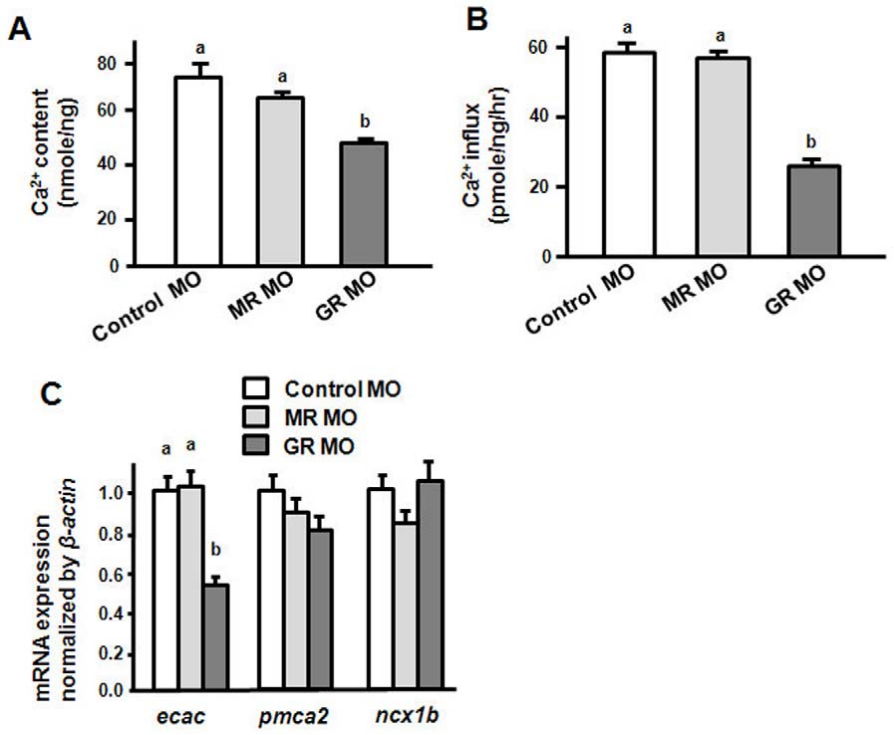
Effects of MR MO and GR MO in 3-dpf zebrafish embryos. Ca^2+^content (A), Ca^2+^ influx (B), and mRNA expressions (C). mRNA expressions were analyzed by qPCR and values were normalized to *β-actin*. ^abc^Indicate a significant difference (*p*<0.05) using Tukey's multiple-comparison test following one-way ANOVA. Values are the mean ± SEM (*n* = 6 or 7).

After specificity tests, respective MOs were injected into 1∼2-cell embryos. Compared to the control MO, the GR MO caused significant increases in Ca^2+^ content and influx in 3-dpf zebrafish embryos, but the MR MO had no effects (Fig. 6A, B). The qPCR assay of the mRNA expressions of Ca^2+^ transporters showed that the GR MO significantly reduced expression of the *ecac*, but did not affect *ncx1b* and *pmca2* mRNA expressions in 3-dpf zebrafish embryos (Fig. 6C).

**Figure 7:**
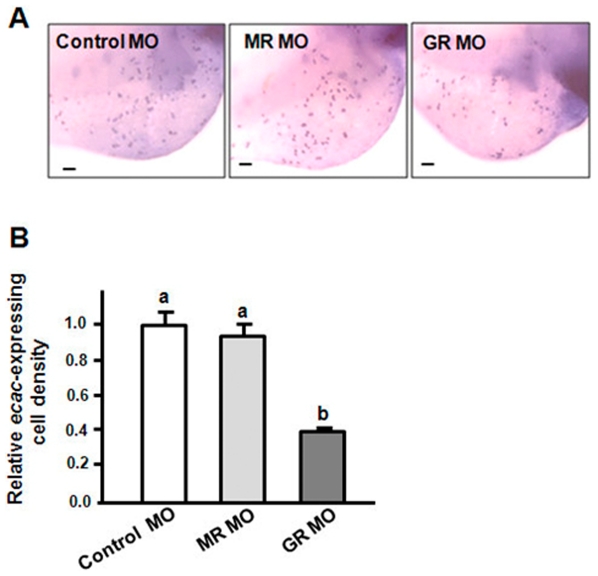
Effects of MR MO and GR MO on *ecac*-expressing cells in 3-dpf zebrafish embryos. In situ hybridization analysis indicated *ecac* signals (A) and density of *ecac*-expressing cells (B). ^abc^Indicate a significant difference (*p*<0.05) using Tukey's multiple-comparison test following one-way ANOVA. Value are the mean ± SEM (*n* = 6 or 7). *Scale bar* 100 µm.

To further support these data, mRNA density of *ecac*-expressing cells in the skin of zebrafish morphants were analysed. The *ecac*-expressing cell was also significantly decreased upon GR MO injection (Fig. 7A, B). In contrast with the GR MO, the MR MO did not affect the expressions of *ncx1b*, *pmca2*, and *ecac* genes as well as the density of *ecac*-expressing cells in 3-dpf zebrafish embryos (Fig. 6C, 7A, B).

### Effects of MR/GR MO on Ca^2+^ influx and *ecac* mRNA expression in zebrafish embryos with exogenous cortisol or low Ca^2+^ media

**Figure 8:**
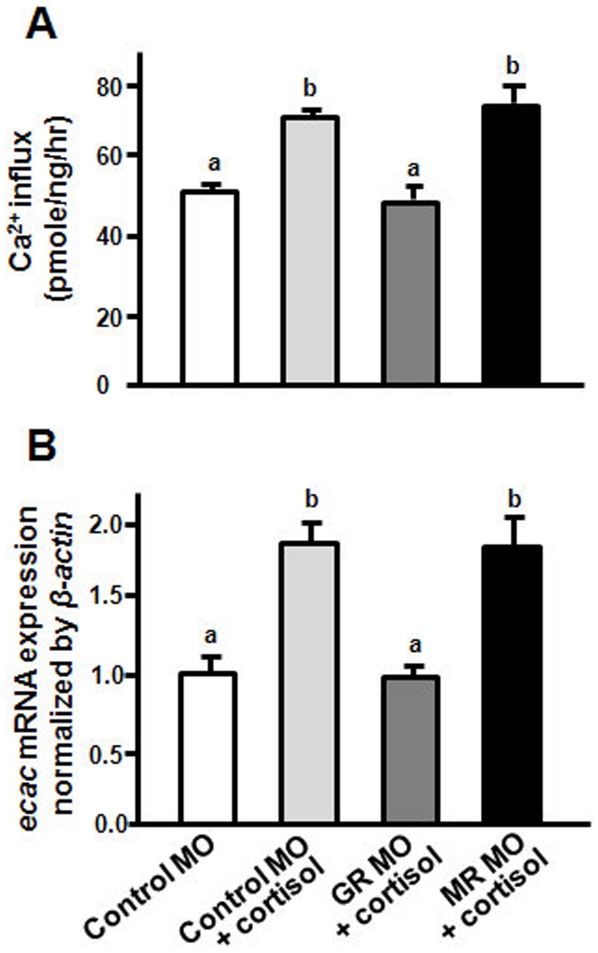
Effects of MR MO and GR MO on zebrafish embryos with cortisol treatment. Ca^2+^ influx (A) and *ecac* mRNA expression (B) were analyzed in 3-dpf zebrafish embryos injected with GR MO or MR MO with cortisol treatment. mRNA expressions were analyzed by qPCR, and values were normalized to *β-actin*. ^abc^Indicate a significant difference (*p*<0.05) using Tukey's multiple-comparison test following one-way ANOVA. Values are the mean ± SEM. (*n* = 6∼8).

To precisely ascertain the different roles of the zebrafish GR and MR, the zebrafish were incubated with or without cortisol (20 mg/l) after injections with the MOs. Compared to the control group (control MO injection without cortisol), both groups of the control MO with cortisol and the MR MO with cortisol exhibited a significantly higher Ca^2+^ influx at 3-dpf, but the GR MO-injected embryos with exogenous cortisol did not (Fig. 8A). Similarly, *ecac* mRNA expression in the control MO with cortisol and MR MO with cortisol was significantly stimulated, while that of the GR MO-injected embryos was not affected by exogenous cortisol (Fig. 8B).

**Figure 9:**
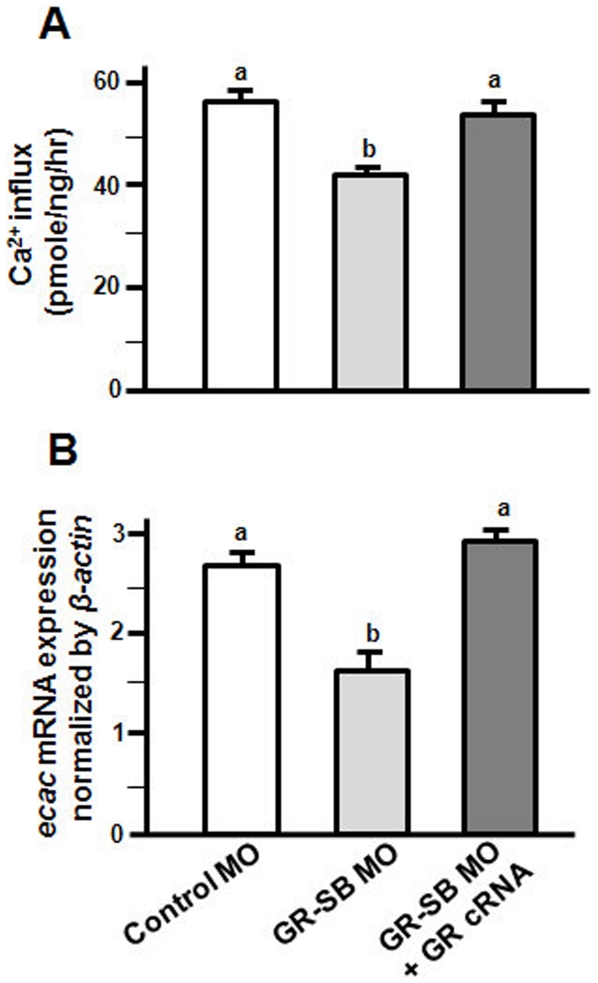
Effects of GR cRNA on GR-SB MO-injected zebrafish embryos. Ca^2+^ influx (A) and *ecac* mRNA expression (B) were also analyzed in 3-dpf zebrafish embryos injected with GR-SB MO or GR-SB MO with GR cRNA. mRNA expressions were analyzed by qPCR, and values were normalized to *β-actin*. ^abc^Indicate a significant difference (*p*<0.05) using Tukey's multiple-comparison test following one-way ANOVA. Values are the mean ± SEM. (*n* = 6∼8).

To further support the role of the GR in the Ca^2+^ uptake mechanism, the zebrafish GR-SB MO and/or GR cRNA were co-injected into zebrafish 1∼2-cell-stage embryos. Compared to the control MO-injected group, GR-SB MO, similar to GR MO, also caused significant decreases in both Ca^2+^ influx and *ecac* mRNA expression in 3-dpf zebrafish embryos (Fig. 9A, B); however, co-injection with zebrafish GR cRNA and the GR-SB MO rescued the defective Ca^2+^ influx and *ecac* mRNA expression caused by the injection of the GR-SB MO (Fig. 9A, B).

**Figure 10:**
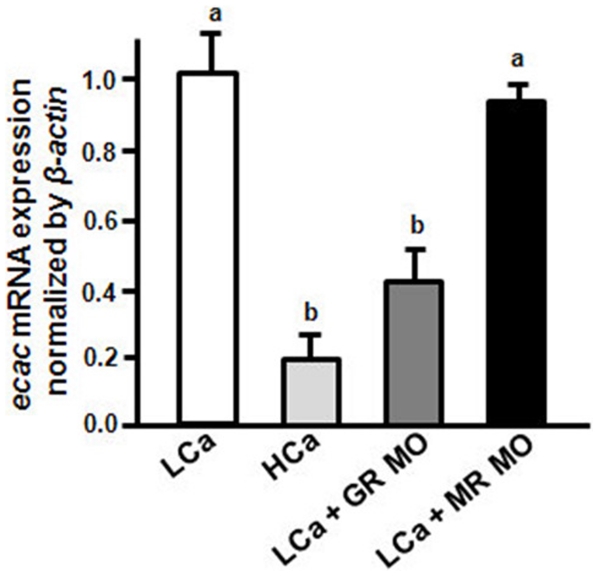
Effect of MR MO and GR MO on *ecac* mRNA expression with low Ca^2+^ treatment. *ecac* mRNA expression were analyzed in 3-dpf zebrafish embryos injected with GR MO or MR MO with low Ca^2+^ (0.02 mM; LCa) treatment. mRNA expressions were analyzed by qPCR, and values were normalized to *β-actin*. ^abc^Indicate a significant difference (*p*<0.05) using Tukey's multiple-comparison test following one-way ANOVA. Values are the mean ± SEM. (*n* = 6∼8).

Low Ca^2+^ medium was known to stimulate *ecac* expression in zebrafish [40], [41] (Fig. 10).Whether this *ecac* expression upregulation by low Ca^2+^ medium is mediated by GR or MR was further clarified in the following experiments. One∼two-cell-stage embryos were injected with the control MO, MR MO, and GR MO, respectively, and then were incubated in 2.0 mM (high) or 0.02 mM (low) Ca^2+^ medium. Compared to the control MO in low Ca^2+^ medium, the GR morphants in low Ca^2+^ medium were significantly lower in the ECaC mRNA expression at 3 dpf, but the MR morphants in low Ca^2+^ medium were similar to the control group (Fig. 10).

### Potential regulatory elements in the zebrafish *ecac* gene

The 5′ flanking region of the zebrafish *ecac* gene was putatively identified and analyzed to search for possible regulatory elements. Several hormone-responsive elements, including the GRE, were identified within the 1653 bp analyzed in the putative upstream promoter region of the *ecac* gene (Fig. S2).

### Exogenous cortisol on mRNA expressions of *ecac* and vitamin D_3_-related genes in zebrafish embryos

**Figure 11:**
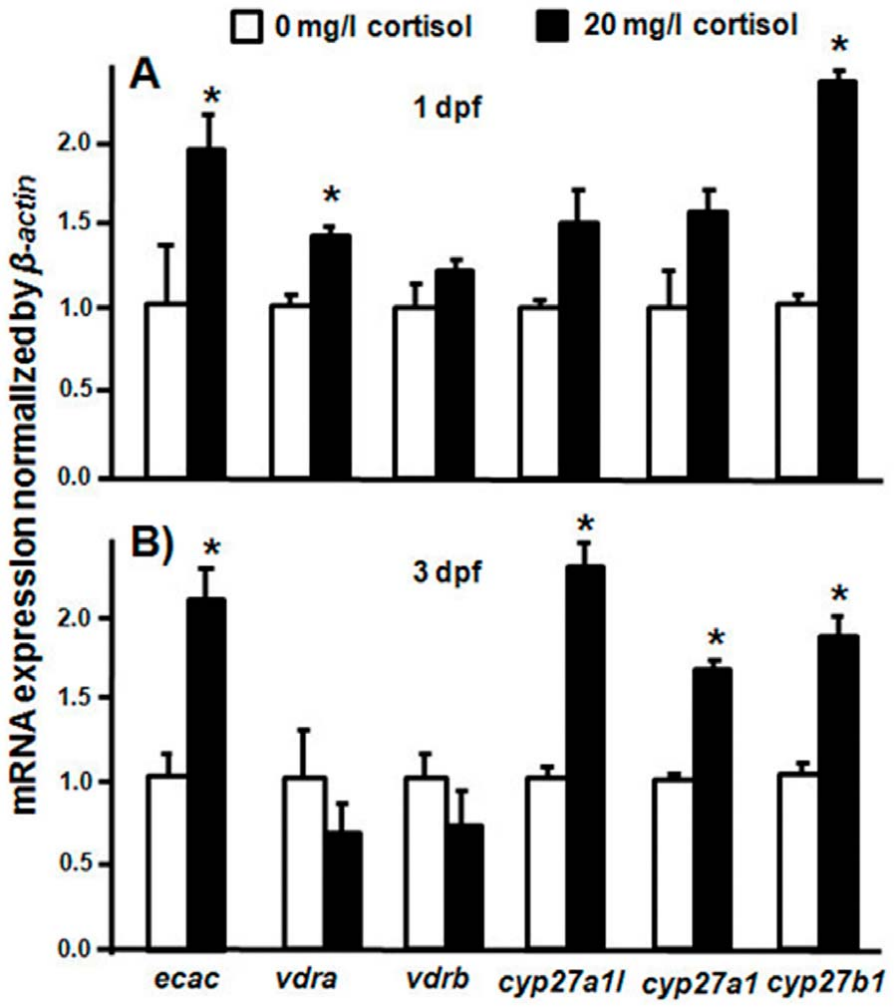
Effects of exogenous cortisol on mRNA expressions of the vitamin D_3_-related genes. qPCR was used to analyze mRNA expression and values were normalized to *β-actin*. (A) mRNA expressions in 1-dpf zebrafish embryos. (B) mRNA expressions in 3-dpf zebrafish embryos. ^abc^Indicate a significant difference (*p*<0.05) using Tukey's multiple-comparison test following one-way ANOVA. Values are the mean ± SEM (*n* = 6).

To investigate the effect of cortisol on the vitamin D_3_-related genes, exogenous cortisol (20 mg/l) was used to treat zebrafish embryos. To trace the effect of exogenous cortisol, 1- and 3-dpf embryos were sampled. Cortisol caused differential effects on mRNA expressions of *ecac* and the vitamin D_3_-related genes in 1-dpf or 3-dpf zebrafish embryos. According to the qPCR, cortisol significantly stimulated *ecac* and *cyp27b1* mRNA expression in 1-and 3-dpf zebrafish embryos; however, cortisol only significantly stimulated the mRNA expressions of *vitamin D_3_ receptor a* (*vdra*), *cyp27a1l* and *cyp27a1*in 1- or 3-dpf embryos (Fig. 11A, B). On the other hand, *vdrb* was not affected by cortisol in 1- and 3-dpf zebrafish embryos (Fig. 11A, B).

### Effects of MR/GR MOs on mRNA expressions of the vitamin D_3_-related genes in zebrafish embryos

**Figure 12:**
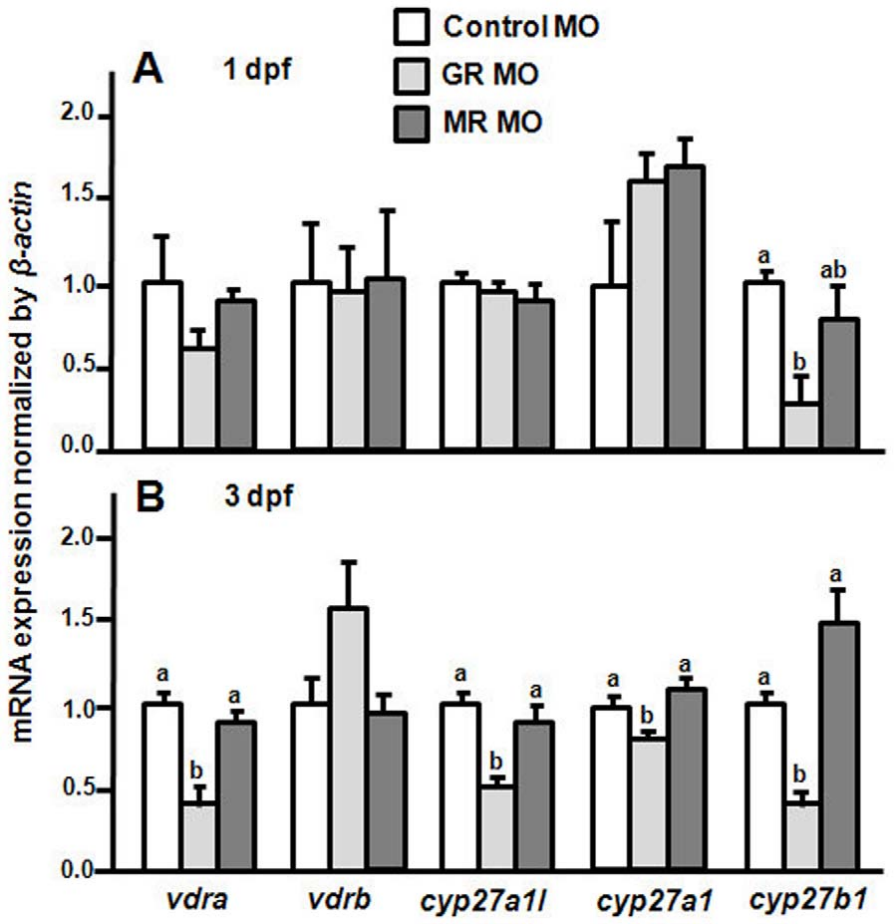
Effects of MR MO and GR MO on mRNA expression of the vitamin D_3_-related genes. (A) mRNA expressions in 1-dpf zebrafish embryos. (B) mRNA expressions in 3-dpf zebrafish embryos. mRNA expression was analyze by qPCR and values were normalized to *β-actin*. ^abc^Indicate a significant difference (*p*<0.05) using Tukey's multiple-comparison test following one-way ANOVA. Values are the mean ± SEM (*n* = 6).

To further support the data of Fig. 7, 1∼2-cell-stage embryos were injected with the control MO, MR MO, and GR MO, respectively. MR MO did not cause any effects on mRNA expressions of the vitamin D_3_-related genes in 1- and 3-dpf zebrafish embryos (Fig. 12A, B). On the contrary, GR MO caused differential effects on those vitamin D_3_-related genes (Fig. 12A, B). Only *cyp27b1* mRNA expression was downregulated by GR MO at 1 dpf (Fig. 12A), but the mRNA expressions of *vdra*, *cyp27a1l* and *cyp27a1* were all decreased by GR MO at 3 dpf (Fig. 12B).

## Discussion

Glucocorticoid (cortisol) showed different impact in Ca^2+^ handling between mammal and fish; however, the understanding is little of cortisol control in fish. For this purpose, we explored cortisol effect in zebrafish Ca^2+^ handling. In the present study, the major findings were the following: (I) expression of zebrafish *11β-hydroxylase* was stimulated by low-Ca^2+^ environment; (II) exogenous cortisol increased zebrafish Ca^2+^ influx and content through upregulating the expression of *ecac* but not those of *ncx1b* or *pmca2*; (III) exogenous cortisol probably through a feedback pathway modulated the mRNA expression of *11β-hydroxylase*, *hsd11b2* and *gr* but not that of *mr* in zebrafish; and (IV) translational knockdown of GR but not MR caused defects in the binding of cortisol or mRNA expression of vitamin D3-related genes, and thus impaired the *ecac* expression and Ca^2+^ uptake function of zebrafish ECaC via the GR, a regulatory pathway that could be mediated by vitamin D_3_.

Fish mainly obtain Ca^2+^ from the aquatic environment with fluctuating Ca^2+^ levels, and therefore the internal Ca^2+^ homeostasis is impacted by environmental Ca^2+^ levels. Fish (at least teleost) bone is acellular, and thus the bone does not provide a pool for Ca^2+^ as it does in terrestrial vertebrates [30]. For internal Ca^2+^ homeostasis and bone formation (particularly in embryonic and larval stages), fish have to regulate the function of Ca^2+^ uptake to cope with a fluctuating environment. Like other teleosts, zebrafish can enhance Ca^2+^ uptake function by stimulating ECaC expression during acclimation to low-Ca^2+^ fresh water [40], [41], [44], and this functional regulation may be associated with cortisol. Flik and Perry [36] reported that acclimation to low-Ca^2+^ fresh water caused an increase in serum cortisol levels in rainbow trout. The present study further explored the mechanism behind this phenomenon. Low-Ca^2+^ fresh water stimulated the mRNA expression of *11β-hydroxylase*, the enzyme in the final step of cortisol synthesis, which suggests that environmental conditions affect steroidogenesis and thus cortisol levels in zebrafish. All these results imply a possible role of cortisol in the control of Ca^2+^ uptake. To test this hypothesis, we treated zebrafish with exogenous cortisol. Similar to trout and eel [37], [46], in zebrafish, exogenous cortisol stimulated the mRNA expression of *ecac* and Ca^2+^ influx, and these functional enhancements resulted in increased Ca^2+^ contents in the whole body (the present study). The present comprehensive data from molecular to the physiological level demonstrated the calciotropic effects of cortisol, and these effects showed a dose-dependent pattern and were of physiological significance.

The ECaC, NCX1b, and PMCA2 are coexpressed in a specific type of ionocyte [41], which achieves the epithelial Ca^2+^ uptake function through the operations of the 3 transporters in zebrafish [32], [33], [39]. Previously, exogenous cortisol was found to stimulate branchial ECaC mRNA and protein expressions in trout [37], but no attempt was made to examine the effects on the other Ca^2+^ transporters (NCX and PMCA). The present study first reports that exogenous cortisol affected *ecac* only but not those of *ncx1b* or *pmca2*; similar result found in cultured gills with exogenous cortisol (Fig. S1). On the other hand, we further directly demonstrated *ecac*-expressing cell density in skin of zebrafish embryos was stimulated by exogenous cortisol. These results supported the previous notion that the ECaC is the rate-limiting step and the gatekeeper channel for active Ca^2+^ transport in fish [39] as in mammals [47]. On the other hand, translational knockdown of stanniocalcin, a hypocalcemic hormone, was reported to stimulate *ecac* expression and Ca^2+^ influx, but not affect the expressions of *ncx1b* or *pmca2* in zebrafish [42]. Similarly in the present study, knockdown of the GR downregulated the expression of *ecac*, but showed no effect on *ncx1b* or *pmca2*. The *ecac* appears to be the major regulatory target gene in response to environmental Ca^2+^ levels and also the upstream control of hormones in zebrafish.

In the present study, exogenous cortisol suppressed the mRNA expression of *11β-hydroxylase* (decreasing the cortisol level) and simultaneously stimulated that of *hsd11b2* (increasing the cortisone level), reflecting a feedback mechanism in controlling levels of corticoids. In mammals, HSD11B2 converts cortisol to cortisone in MR-specific tissues, and this prevents cortisol binding to the MR in these tissues and thus allows aldosterone to bind to the MR [48]. In fish, HSD11B2 can also convert cortisol to cortisone [49], [50]. Thus, stimulation of *hsd11b2* by exogenous cortisol treatment may be a feedback to control cortisol levels in zebrafish. This notion was further supported by the data of *11β-hydroxylase* and the *gr*; gene expressions of zebrafish *11β-hydroxylase* and the *gr* were inhibited by exogenous cortisol treatment. In mammals, dexamethasone treatment suppressed the secretion of the adrenocorticotrophic hormone, resulting in a decline in serum corticosterone levels [51], [52], and the duodenal and renal *gr* expressions were significantly downregulated by dexamethasone treatment [6]. Similarly in trout and salmon, cortisol treatment by infusion, feeding, or soaking also caused downregulation of CS receptors or the *gr* in gills and liver [53]–[57]. Taken together, cortisol may regulate the function of the Ca^2+^ mechanism through sophisticated feedback pathways, in which the expressions of *11β-hydroxylase*, *hsd11b2*, and *CR*s are differentially modulated.

Physiological functions of cortisol signaling are mediated by the GR and MR, which are ligand-activated transcription factors. In many previous studies, exogenous cortisol treatment was found to stimulate Ca^2+^ uptake in teleosts [36], [38], [46]; however, it was unknown until the present study that cortisol controls the Ca^2+^ uptake function through the GR but not the MR. In addition to experiments of exogenous cortisol treatment, we used a gene-specific MO to abolish endogenous cortisol signaling in zebrafish and directly explored the effect of cortisol-signaling defects on the Ca^2+^ uptake function. Translational knockdown of the MR did not affect Ca^2+^ uptake in zebrafish, but GR knockdown evidently impaired the Ca^2+^ uptake mechanism by decreasing both Ca^2+^ influx and content. Moreover, these defects in the Ca^2+^ uptake mechanism were due to suppression of *ecac* expression but not the expressions of *ncx1b* or *pmca2*. GR defect was also demonstrated to downregulate *ecac* -expressing cell density in skin of zebrafish. These results indicated that the target of the GR is the *ecac*. Interestingly, the Ca^2+^ influx, *ecac* mRNA and *ecac* -expressing cell density in MR MO-injected zebrafish morphants could still be stimulated (compared to the control) by exogenous cortisol, suggesting that the effects of exogenous cortisol on Ca^2+^ uptake function is not through the MR. On the other hand, exogenous cortisol did not cause further stimulation (compared to the control) in Ca^2+^ influx or *ecac* mRNA in GR morphants, but successfully rescued the Ca^2+^ uptake functional defects caused by the GR MO. Similar effect was also found in GR MO-injected zebrafish with low Ca^2+^ freshwater. GR MO morphant can abolish stimulation of low Ca^2+^ freshwater on *ecac* mRNA expression, but MR MO morphant can not. Reinforcing these results, overexpression of the GR by injection with GR cRNA rescued the Ca^2+^ uptake mechanism that was impaired in GR MO morphants. This evidence strongly suggests that endogenous cortisol stimulates Ca^2+^ uptake through the GR, but not the MR, in zebrafish.

Some previous in vitro studies indicated that the trout MR and GR could bind cortisol and stimulate transcriptional activity in the mammalian cell lines transfected with a reporter plasmid [12]–[15]. In a recent study on Atlantic salmon, Kiilerich et al. [16] used GR and MR antagonists to discover that the 2 CS receptors were involved in regulating various ion transporters (NKA, NKCC, and CFTR) during acclimation to salinity changes. Differences between salmon (involvement of both the GR and MR) and zebrafish (only the GR) may be because of differences in the ion transport functions (Na^+^/Cl^−^ vs. Ca^2+^) and species (euryhaline vs. stenohaline), and clarification of this point requires further studies. On the other hand, it was noted that zebrafish MR expression was not regulated by exogenous cortisol in the present study. Teleosts might not synthesize aldosterone, and DOC was proposed to play a similar role as aldosterone [15]. Moreover, DOC was suggested to be a potent agonist of the fish MR because DOC can induce greater transcription activity than cortisol through the trout MR expressed in a mammalian cell line co-transfected with a reporter plasmid [15]. It will be challenging to see if a lack of an effect of exogenous cortisol on MR expression is due to different ligand affinities between the 2 CS receptors in zebrafish.

Cortisol shows hypercalcemic effects in zebrafish and other fish species as described above. On the contrary, cortisol was reported to inhibit the intestinal Ca^2+^ absorption in chickens [58], and GC drugs, dexamethasone and prednisolone, were demonstrated to inhibit duodenal Ca^2+^ uptake and *trpv6* (*ecac*) gene expression in mice [5], [6]. Recently, Kim et al. [6] found only one GRE in the promoter region of mice *trpv6*, and suggested that dexamethasone might not directly regulate *trpv6* transcription, but instead, downregulates the Ca^2+^ uptake function through other hormones. Based on a bioinformatics analysis, there are some putative GREs in the promoter region of the zebrafish *ecac* (Fig. S2). Previous in situ hybridization study on developing zebrafish embryos [59], *gr* transcript was detected over the skin where ionocytes appear. As shown in Fig. S3, GR was colocalized in NaR cells, which express ECaC [40]. These imply the possibility that cortisol can stimulate mRNA expression of the *ecac* through interaction with the GREs; however, further studies are needed to support this notion.

In addition to direct regulation of *ecac* transcript, cortisol may modulate the other endocrine to affect Ca^2+^ uptake in zebrafish. Vitamin D_3_ is a well-known calcitrophic endocrine to regulate Ca^2+^ homeostasis in vertebrates [19]–[21], and several mammal studies demonstrated that glucocorticoid could affect vitamin D_3_ metabolism [24]–[29]. Vitamin D_3_ can bind VDR, forming a vitamin D3-VDR complex. This complex directly stimulates mammalian intestinal *ecac* transcript by binding vitamin D_3_ responsive element (VDRE) in the promoter region of *ecac* [18]. In fish, vitamin D_3_ was also reported to elevate the serum Ca^2+^ level [20], [21], and putative VDRE was also identified in *ecac* promoter region [60] (Fig. S2). Recent in vitro study in medaka, VDR was found to simulate transcript level of VDRE-containing construct with 1,25(OH)_2_D_3_ treatment [61]. Our unpublished data also indicated stimulation of *ecac* mRNA expression by1,25(OH)_2_D_3_ in zebrafish embryos. Taken together, vitamin D3-VDR control of *ecac* expression and function may also exist in fish. However, the associations between cortisol, vitamin D_3_ and Ca^2+^ handling in fish were not clear until the present study. CYP27A1 is an enzyme to synthesize 25(OH)D_3_, the vitamin D_3_ precusor, and subsequently 25(OH)D_3_ is converted to 1,25(OH)_2_ D_3_ (ctive vitamin D_3_) by CYP27B1 [17]. In zebrafish embryos, exogenous cortisol could upregulate the expressions of *cyp27b1*, *cyp27a1* and/or *cyp27a1 like* (*cyp27a1l*) at 1- and 3 dpf, and also affected *vdra* mRNA expression at 1 dpf. These implied that cortisol starts affecting the vitamin D_3_-VDR signaling from early stage. Cortisol could not only stimulate synthesis of vitamin D_3_ precursors but also accelerate the synthesis of active vitamin D_3_. Moreover, our subsequent knockdown experiment further reinforced this notion. Knockdown of GR, but not MR, was found to suppress the expressions of *cyp27b1*, *cyp27a1*, *cyp27a1l* and *vdra* at 1- and/or 3 dpf. According to these results, we suggested that cortisol probably regulates Ca^2+^ handling through vitamin D_3_-VDR system, besides directly regulating *ecac* expression.

In this study, cortisol was suggested to directly or indirectly stimulate Ca^2+^ uptake in zebrafish. Cortisol appears to cause different effects on Ca^2+^ uptake between teleosts and higher vertebrates. Sources of Ca^2+^ fundamentally differ among vertebrates. For terrestrial vertebrates, food is the major source of Ca^2+^, and the intestines serve as a primary site for Ca^2+^ uptake. However, fish are constantly facing aquatic environments with variable Ca^2+^ concentrations (of as low as <0.01 mM in soft fresh water), and gills or the skin serve as the primary site for Ca^2+^ uptake [30], [32], [33]. These differences may be associated with different modes of cortisol control of Ca^2+^ uptake, which is a challenging issue to be explored from an evolutionary point of view.

## Supporting Information

Table S1 **Primers for the RT-PCR analysis.**
(DOC)

Table S2 **Primers for the qPCR analysis.**
(DOC)

Table S3 **Primers for cRNA expression cloning.**
(DOC)

Figure S1 **Effect of exogenous cortisol on mRNA expression of** ***ecac*** **,** ***pmca2*** **and** ***ncx1b*** **in cultured gills.** *ecac* mRNA expression was analyzed by qPCR and values were normalized to *β-actin*. ^abc^Indicate a significant difference (*p*<0.05) using Tukey's multiple-comparison test following one-way ANOVA. Values are the mean ± SEM (*n* = 5).
(TIF)

Figure S2 **Upstream regulatory region of the zebrafish** ***ecac*** **gene.** The transcription initiation sites are marked by +1, and the start codon (ATG) is marked by a square. The putative upstream regulatory elements are underlined. The core sequence of each element is shown in bold font. GRE, glucocorticoid-responsive element; VDRE, vitamin D_3_-responsive element; ARE, androgen-responsive element.
(TIF)

Figure S3 **Co-localization of** ***gr*** **mRNA by in situ hybridization with anti-NKA using immunocytochemical analysis of zebrafish gill cryosections.** (A) in situ hybridization of *gr* mRNA; (B) immunocytochemical staining of NKA. Arrow indicated *gr* mRNA and NKA protein signals at similar area. *Scale bar* 5 µm.
(TIF)
