# Supplementary material for: Reverse Effect of Mammalian Hypocalcemic Cortisol in Fish: Cortisol Stimulates Ca2+ Uptake via Glucocorticoid Receptor-Mediated Vitamin D3 Metabolism
Source: PLoS One. 2011 Aug 24;6(8):e23689. doi: 10.1371/journal.pone.0023689 (PMC3161063; doi:10.1371/journal.pone.0023689)
Supplement: Table S1 — Primers for the RT-PCR analysis. (DOC) [file pone.0023689.s001.doc]

**Table S**1 Primers for the RT-PCR analysis

| Gene name |  | Primer sequence |
| --- | --- | --- |
| *gr* | F | 5' ACAGCTTCTTCCAGCCTCAG 3' |
|  | R | 5' CCGGTGTTCTCCTGTTTGAT 3' |
| *mr* | F | 5' ACAGAGGCAACAATGATTAGAG 3' |
|  | R | 5' GTTCTCCCACAAAGAGGGT 3' |
| *β-actin* | F | 5' ATTGCTGACAGGATGCAGAAG 3' |
| F:Fordward, R:Reverse. | R | 5' GATGGTCCAGACTCATCGTACTC ' |
