# Supplementary material for: Reverse Effect of Mammalian Hypocalcemic Cortisol in Fish: Cortisol Stimulates Ca2+ Uptake via Glucocorticoid Receptor-Mediated Vitamin D3 Metabolism
Source: PLoS One. 2011 Aug 24;6(8):e23689. doi: 10.1371/journal.pone.0023689 (PMC3161063; doi:10.1371/journal.pone.0023689)
Supplement: Table S2 — Primers for the qPCR analysis. (DOC) [file pone.0023689.s002.doc]

**Table S2 Primers for the quantitative q**PCR analysis

| Gene name |  | Primer sequence | |
| --- | --- | --- | --- |
| *ecac* | F | 5' TCCTTTCCCATCACCCTCT 3' | |
|  | R | 5' GCACTGTGGCAACTTTCGT 3' | |
| *pmca2* | F | 5' AAGCAGTTCAGGGGTTTAC 3' | |
|  | R | 5' CAGATCATTGCCTTGTATCA3' | |
| *ncx1b* | F | 5' TAAAGTGGCAGCGATACAGGT 3' | |
|  | R | 5' CAGATCAAGGCGAAGATGG3' | |
| *gr* | F | 5' ACAGCTTCTTCCAGCCTCAG 3' | |
|  | R | 5' CCGGTGTTCTCCTGTTTGAT 3' | |
| *mr* | F | 5' ACAGAGGCAACAATGATTAGAG 3' | |
|  | R | 5' GTTCTCCCACAAAGAGGGT 3' | |
| *11β-hydroxylase* | F | 5' ATCGAGAGACACGCAGACAC3' | |
|  | R | 5'ACAGACGAGGACACCATCAC3' | |
| *hsd11b2* | F | 5' TTGGTGGAGCAGTGAAGAAG 3' | |
|  | R | 5' CACAGGGCACACAGTCTCTC 3' | |
| *vdra* | F | 5' CTCGGATTCTGTGGATGCTT 3' | |
|  | R | 5' GGCCTTACGCTTCATACTGC 3' | |
| *vdrb* | F | 5' ACACAGCGTGGAGTGGAGT 3' | |
|  | R | 5' ACACTCCATGGCAAGAACA 3' | |
| *cyp27a1* | F | 5' CCGCAAATATTTCCCATTCT 3' | |
|  | R | 5' CTCCAGCAACATCCTGATTG 3' | |
| *cyp27a1l* | F | 5' AGCGCATCAGAAGCATATTG 3' | |
|  | R | 5' CATCATTGCCTTTGGTTGTC 3' | |
| *cyp27b1* | F | 5' TCTATCCTGTTATTCCAGCCAA 3' | |
|  | R | 5' GCCTGAAGGAGTCTGGATCT 3' | |
| *b-actin* | F | 5' ATTGCTGACAGGATGCAGAAG 3' | |
|  | R | 5' GATGGTCCAGACTCATCGTACTC 3' | |
|  | | |  |

The accession numbers of nucleic acid sequences are as followed: *ecac* [GenBank:NM_001001849], *pmca2* [GenBank:NM_001123238], *ncx1b* [GenBank:NM_001039144], *gr* [GenBank:NM_001020711], *mr* [GenBank:NM_001100403], *11β-hydroxylase* [GenBanK:DQ650710] , *hsd11b2* [GenBank:NM_212720], *vdra* [GenBank:NM_130919], *vdrb* [GenBank:NM_001159985], *cyp27a1l* [GenBank:XM_001333968], *cyp27a1* [EMBL:ENSDARG00000057262], *cyp27b1* [EMBL:ENSDART00000066178], *β-actin* [GenBank:NM_181601]
