# Supplementary material for: Reverse Effect of Mammalian Hypocalcemic Cortisol in Fish: Cortisol Stimulates Ca2+ Uptake via Glucocorticoid Receptor-Mediated Vitamin D3 Metabolism
Source: PLoS One. 2011 Aug 24;6(8):e23689. doi: 10.1371/journal.pone.0023689 (PMC3161063; doi:10.1371/journal.pone.0023689)
Supplement: Table S3 — Primers for cRNA expression cloning. (DOC) [file pone.0023689.s003.doc]

**Table S**3 Primers for cRNA expression cloning

| Name |  | Primer sequence |
| --- | --- | --- |
| *GR/PCS2+XLT* | F | 5' CCGGATCCTGCAAAATGGATCAAGGAG 3' |
|  | R | 5' CCTCTAGAGAAGGGGACCAAGGTCTG 3' |
| *MR/PCS2+XLT* | F | 5' CCGGATCCGGTATGGAGACTAAAAGA 3' |
|  | R | 5' CCTCTAGATGAGTCT GTTTCTGACTT 3' |
| *GR/PCS2+* | F | 5' GAAGGATCCTCAGACTGCG 3' |
|  | R | 5' AGCTCTAGATGAAACTCTGCG ' |
