# Supplementary figures and images for: Reverse Effect of Mammalian Hypocalcemic Cortisol in Fish: Cortisol Stimulates Ca2+ Uptake via Glucocorticoid Receptor-Mediated Vitamin D3 Metabolism
Source: PLoS One. 2011 Aug 24;6(8):e23689. doi: 10.1371/journal.pone.0023689 (PMC3161063; doi:10.1371/journal.pone.0023689)

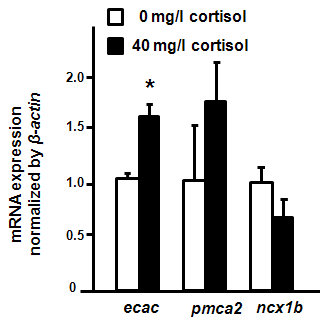

Supplement: Figure S1 — Effect of exogenous cortisol on mRNA expression of ecac , pmca2 and ncx1b in cultured gills. ecac mRNA expression was analyzed by qPCR and values were normalized to β-actin. abcIndicate a significant difference (p<0.05) using Tukey's multiple-comparison test following one-way ANOVA. Values are the mean ± SEM (n = 5). (TIF) [file pone.0023689.s004.tif]

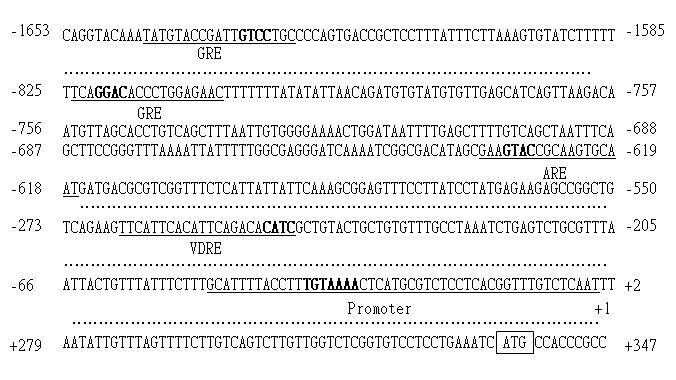

Supplement: Figure S2 — Upstream regulatory region of the zebrafish ecac gene. The transcription initiation sites are marked by +1, and the start codon (ATG) is marked by a square. The putative upstream regulatory elements are underlined. The core sequence of each element is shown in bold font. GRE, glucocorticoid-responsive element; VDRE, vitamin D3-responsive element; ARE, androgen-responsive element. (TIF) [file pone.0023689.s005.tif]

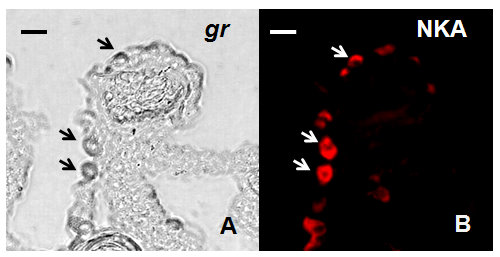

Supplement: Figure S3 — Co-localization of gr mRNA by in situ hybridization with anti-NKA using immunocytochemical analysis of zebrafish gill cryosections. (A) in situ hybridization of gr mRNA; (B) immunocytochemical staining of NKA. Arrow indicated gr mRNA and NKA protein signals at similar area. Scale bar 5 µm. (TIF) [file pone.0023689.s006.tif]
